# Does a novel 3D printed individualized guiding template based on cutaneous fiducial markers contribute to accurate percutaneous insertion of pelvic screws? A preliminary phantom and cadaver study

**DOI:** 10.1186/s12893-024-02402-6

**Published:** 2024-04-13

**Authors:** Xingguang Tao, Fei Lyu, Kapil Sugand, Kaihua Zhou, Huixiang Wang

**Affiliations:** 1 Department of Orthopedics, Qingpu Branch of Zhongshan Hospital, Fudan University, Shanghai, China; 2 Department of Orthopedics, Affiliated Hospital of Yangzhou University, Yangzhou University, Yangzhou, China; 3 Royal National Orthopaedic Hospital, Stanmore, UK; 4 Department of Orthopedics, Shanghai Sixth People’s Hospital Affliated to Shanghai Jiao Tong University School of Medicine, Shanghai, China

**Keywords:** Pelvic fracture, Bone screws, Fiducial markers, 3D Printing, Personalized guiding template

## Abstract

**Background:**

Most 3D-printed guiding templates require dissection of soft tissues to match the corresponding surfaces of the guiding templates. This study sought to explore the accuracy and acceptability of the novel 3D printed individualized guiding templates based on cutaneous fiducial markers in minimally invasive screw placement for pelvic fractures.

**Methods:**

The printed template was tested on five high-fidelity biomimetic phantom models of the bony pelvis and its surrounding soft tissues as well as on two fresh frozen cadavers. Four cutaneous fiducial markers were transfixed on each phantom model prior to performing CT scans to reconstruct their 3D models. Personalized templates for guiding screw insertion were designed based on the positions of the fiducial markers and virtually planned target screw channels after scanning, followed by 3D printing of the guide. Phase 1 consisted of five expert surgeons inserting one anterograde supra-pubic screw and one sacroiliac screw percutaneously into each phantom model using the 3D-printed guide. The deviation of screw positions between the pre-operative planned and post-operative actual ones was measured after registering their 3D modelling. A Likert scale questionnaire was completed by the expert surgeons to assess their satisfaction and acceptability with the guiding template. Phase 2 consisted of repeating the same procedures on the fresh frozen cadavers in order to demonstrate face, content and concurrent validity.

**Results:**

In Phase 1, all ten screws were successfully implanted with the assistance of the guiding template. Postoperative CT scans confirmed that all screws were safely positioned within the bony pelvic channels without breaching the far cortex. The mean longitudinal deviation at the bony entry point and screw tip between the pre-operative planned and post-operative actual screw paths were 2.83 ± 0.60 mm and 3.12 ± 0.81 mm respectively, with a mean angular deviation of 1.25 ± 0.41°. Results from the Likert questionnaire indicated a high level of satisfaction for using the guiding template among surgeons. In Phase 2, results were similar to those in Phase 1.

**Conclusions:**

The 3D-printed guiding template based on cutaneous fiducial markers shows potential for assisting in the accurate insertion of percutaneous screws in the pelvis.

## Introduction

Unstable pelvic fractures (AO/OTA Type B and C) with anterior and posterior ring injuries often require surgical fixation [1–4]. Compared with fixation methods such as external fixation and open reduction and internal fixation (ORIF) with plating, percutaneous pelvic screw fixation is relatively more stable biomechanically [5, 6]. The latter is highly recommended due to being least invasive with shorter recovery. However, insertion of a pelvic screw is technically challenging as it has a narrow safe bone channel resulting in higher risks of iatrogenic and intraoperative complications such as neurovascular injury, revision, and long term disability. Traditional percutaneous screw insertion mainly relies on repeated fluoroscopy to confirm satisfactory positioning, which increases intraoperative radiation for both the patient and operating staff and has a prolonged learning curve. The navigation system can provide good accuracy in the precise implantation of screws. It uses infrared to register and track the pelvis and operating instruments during surgery, guiding pelvic screws to be implanted along the path, and the operation is not complicated. The navigation system is the most competitive but still only equipped in few hospitals. In amidst the digital revolution, the clinical application of 3D printed patient-specific guiding templates provides a new and promising method for accurate insertion of percutaneous screws [7].

Most current 3D printed guiding templates require dissection of soft tissues planes down to exposed bony surfaces to match the corresponding surfaces of the guiding templates [7–9]. However, the soft tissues around the pelvis are thick and adjacent to vital neurovascular bundles, making this traditional guiding template unsuitable for percutaneous pelvic screw insertion. In recent years, studies have adopted 3D printed guiding templates based on cutaneous fiducial markers to assist percutaneous screw fixation [10, 11]. Pre-operative marking is performed on the thinnest aspect of the skin-bone interface followed by application of the fiducial markers to design the principal component of the guiding template. Subsequently, the guide based on the virtually planned target screw channel is combined with the principal component. Consequently, these 3D printed guiding templates based on cutaneous fiducial markers do not require dissection of soft tissues to expose the bony surface for matching, thus having the advantage of non-invasiveness compared to traditional guiding templates. However, to the best of our knowledge, there are no reports of individualized non-invasive guiding templates based on skin markers for pelvic fracture fixation yet.

### Aim

This pilot study was the first trial of the application of novel non-invasive 3D printed guiding templates using five high-fidelity pelvic phantom models and two fresh frozen cadavers to guide the insertion of percutaneous pubic screws and sacroiliac screws.

### Objectives

Primary objective was to explore the accuracy of the novel 3D printed individualized guiding templates in minimally invasive screw placement for pelvic fractures. Secondary objective was to observe for validity and acceptability by the expert Attending Orthopaedic Surgeons participating in the study.

## Materials and methods

### Phase 1: phantom models

#### External marking on the pelvic phantom models and data acquisition

**Fig. 1:**
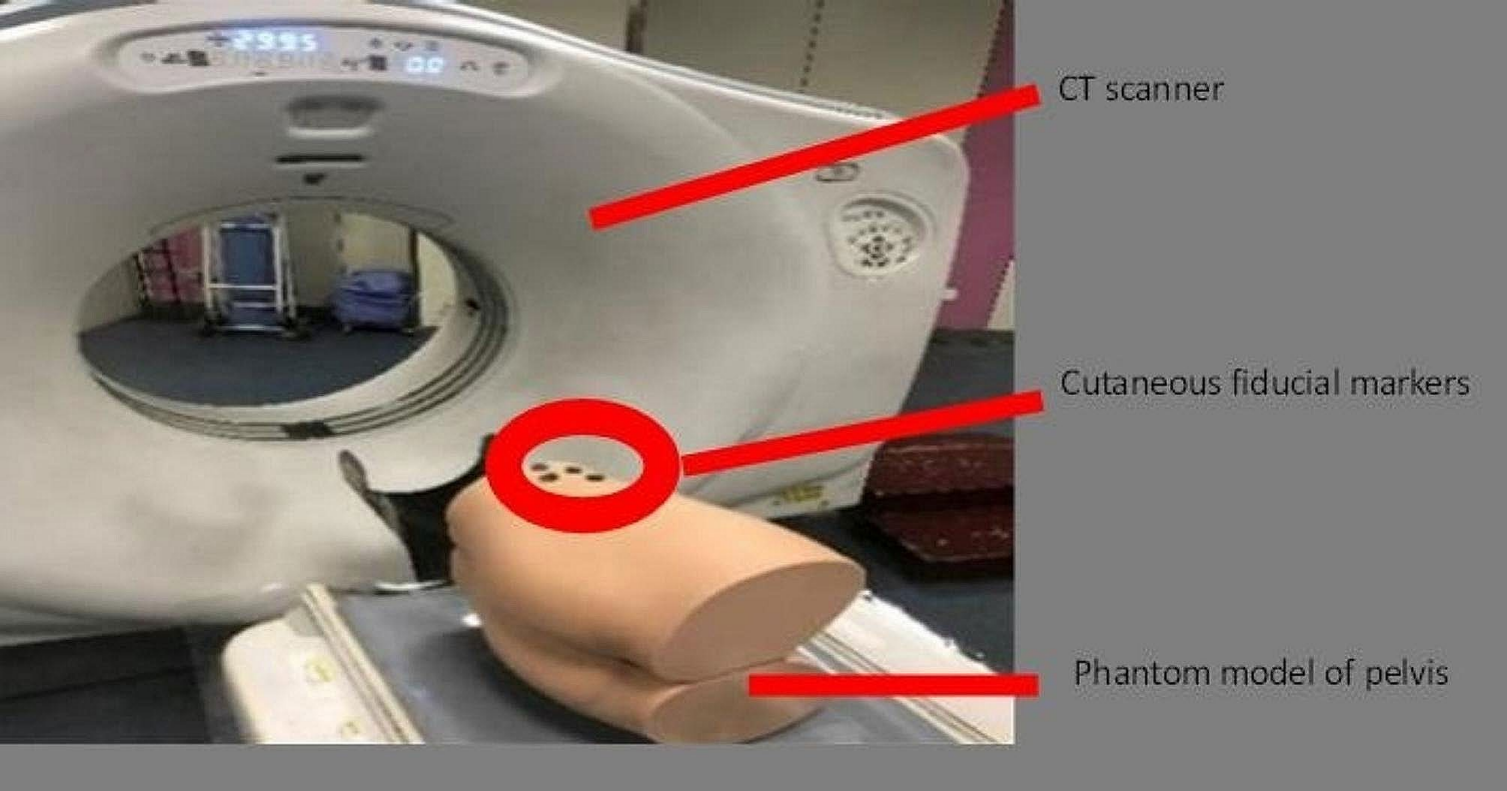
CT scan of the pelvic high-fidelity simulation phantom model with four fiducial makers pasted on the skin overlying the iliac crest (i.e. thinnest skin-bone interface)

Five high-fidelity biomimetic pelvic simulation phantom models with sawbone and soft tissue coverage (Shanghai Sunshine Medical Technology Company, Shanghai, China; Fig. 1) were used. The soft tissues included the skin and subcutaneous tissue. They are printed by photosensitive resins. The morphology of the sawbone and soft tissue envelope in these phantom models were 3D printed based on the data of the Chinese Digital Human. They were made of biomimetic material and manufactured to highly simulate real human anatomy visually and tangibly, mimicking the mechanical properties from data from cadaveric testing conducted at Shanghai Jiaotong University. The pelvic bone structures in the phantom models did not contain fractures or dislocations, in order to simulate pelvic fractures after reduction or without obvious displacement.

Four external fiducial markers were securely attached to the skin surface of the iliac crest using Compont medical glue (Compont Company, Beijing, China). Subsequently, a CT scan was performed to collect pelvic data with the markers firmly affixed to the skin (Fig. 1). The scanning conditions consisted of voltage 120 kV, current 150 mA, and layer thickness 0.625 mm. The collected CT data was stored in DICOM format.

### Design of safe bony channels for screw insertion

The CT data in DICOM format was imported into Mimics software (version 16.0, Materialise, Leuven, Belgium), and the 3D pelvic model was reconstructed. The bony, surface skin morphology, and fiducial marker masks were denoised separately through software threshold adjustment, region growth, manual segmentation, and merging functions. Subsequently, the masks were individually 3D reconstructed to obtain 3D models of the pelvic bone and the surface skin with the external markers attached.

After obtaining the CT reconstructed 3D models in Mimics software, the screw placement channels were virtually planned, and the ideal screw lengths were determined and recorded. The desired characteristics of the safe bony channels consisted of the three criteria: (i) ensuring that the entire pathway remained within the bone medullary passage and as far away from the bony cortex as possible, (ii) ensuring that the supra-pubic screw path did not penetrate intra-articularly into the acetabulum or extend beyond the pubic bony far cortex, and (iii) ensuring that the sacroiliac screw path did not penetrate into the sacral hiatus, sacral canal or extend beyond the anterior sacral bony cortex. In Mimics, a 2.5 mm diameter cylinder was used to simulate the guiding needle in order to determine the safe bony channel. Subsequently, the 3D models of the pelvic bone, the skin surface with the external fiducial markers, and the screw channels were exported and saved in STL format.

### Design and 3D printing of the guiding template

The guiding template comprised of two parts: (i) the main body that matched the cutaneous fiducial markers, and (ii) the guiding director that facilitated needle insertion. Geomagic Studio 12.0 software (Geomagic, Rock Hill, SC, USA) was utilized to import the 3D models saved in STL format. The design of the guiding template was based on the spatial position of the four circular external fiducial markers and the skin surface morphology. The main body of the guiding template was created with four holes embedded at the marker points, and the inner surface of the main body conformed to the skin morphology.

**Fig. 2:**
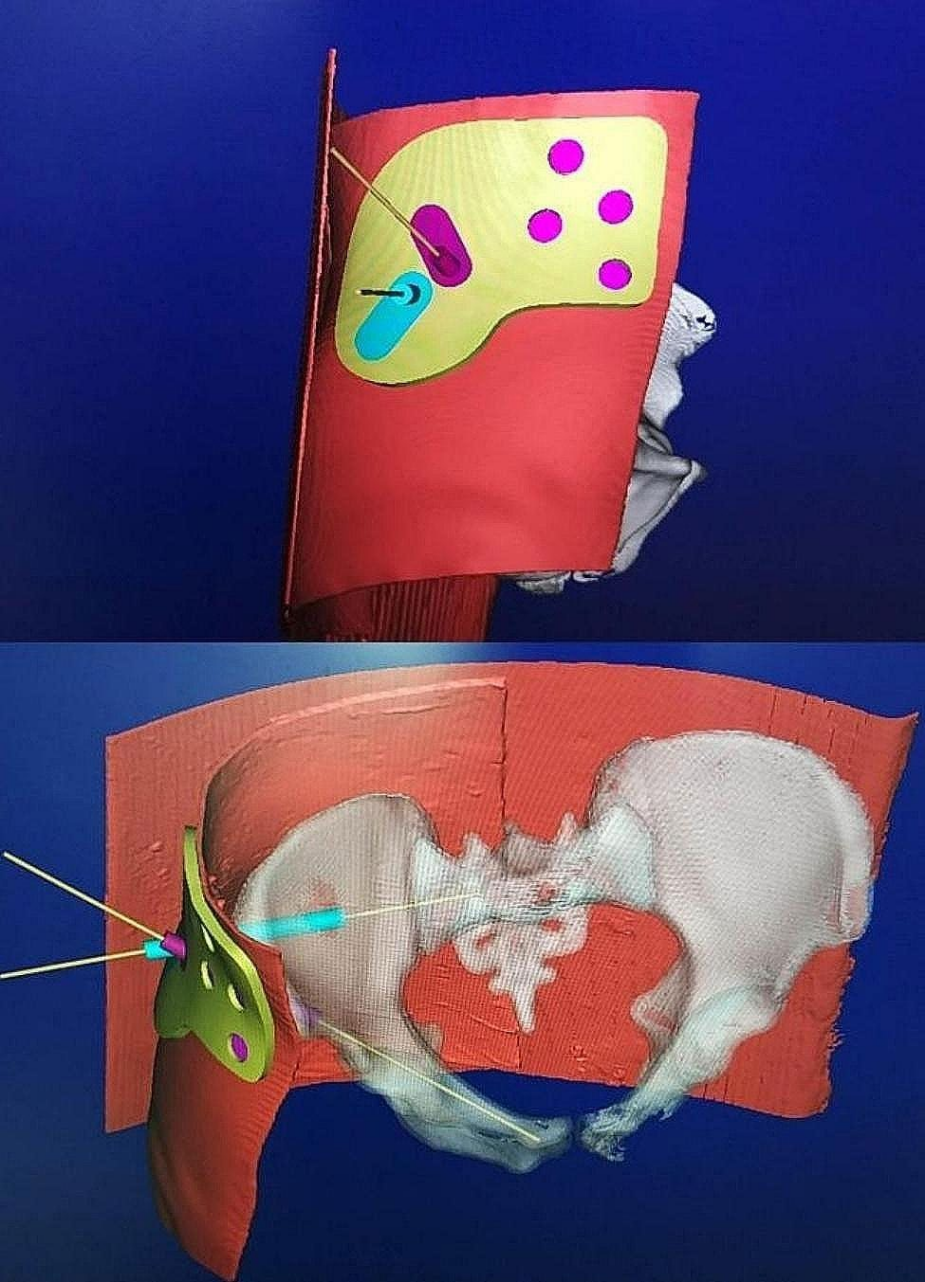
Design of the main body (white arrow) of the guiding template and two guiding directors (black arrows) for the suprapubic and sacroiliac screws based on the 3D positions of the external fiducial markers, skin morphology and the planned virtual screw channel from imaging

**Fig. 3:**
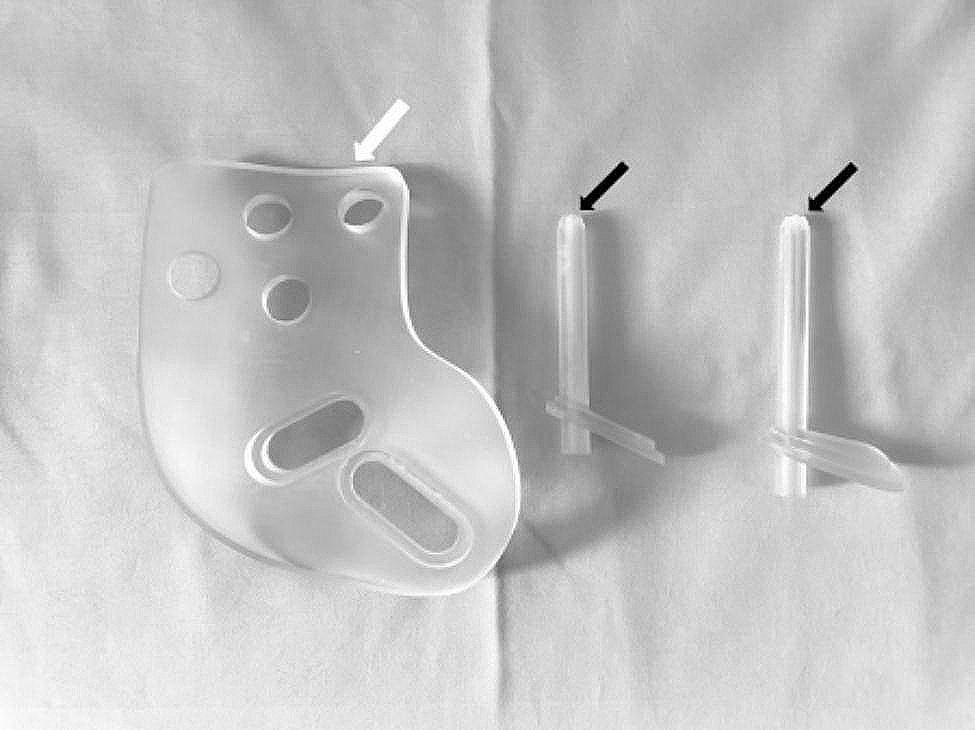
3D printed guiding templates consisting of one main body part and two guiding directors (for suprapubic and sacroiliac screws respectively). The tip of the cannulated cylinder was designed with saw tooth and gradually thinner (black arrows) to improve the contact with near bony cortex, control of placement and stability during the operation

By extending the virtual screw channel in reverse, the guiding director was designed to be coaxial with an inner diameter of 2.8 mm and an outer diameter of 6.0 mm, allowing it to fit the guiding needle utilized in surgery. The guiding director took the form of a cannulated cylinder, with the tail part designed with a connecting structure to be embedded in the main body of the guiding template. The tip of the cannulated cylinder was designed with saw teeth in order to make contact with the bone, simulating the insertion of the guiding director into soft tissue and providing direct support against the bony surface. This enhanced and maintained the stability of the guiding template during the operation. Separate guiding directors were designed for the suprapubic and sacroiliac screws respectively, based on their respective virtual screw channels. However, a single main body was used for both the suprapubic and sacroiliac guiding directors. Consequently, the guiding template consisted of one main body for matching the skin markers and two separate guiding directors for the suprapubic and sacroiliac screws respectively (Fig. 2). Lastly, the designed guiding template model was 3D printed using photosensitive resin material (Fig. 3).

### Experimental operation on phantom

The high-fidelity biomimetic pelvic simulation phantom model was positioned on the operating table, and the main part of the 3D printed guiding template was securely aligned with the cutaneous fiducial markers. A 15 mm incision was made on the cutaneous layer only, and the guiding director for the sacroiliac screw was inserted into the main body of the guiding template. The participating expert Attending Orthopaedic Surgeons ensured a press fit between the main body and the guiding director. The tip of the guiding director was inserted through the incision and pressed against the surface of the iliac bone, while the tail part was firmly matched with the main body. Simultaneously, the alignment between the main part of the template and the four markers was maintained.

**Fig. 4:**
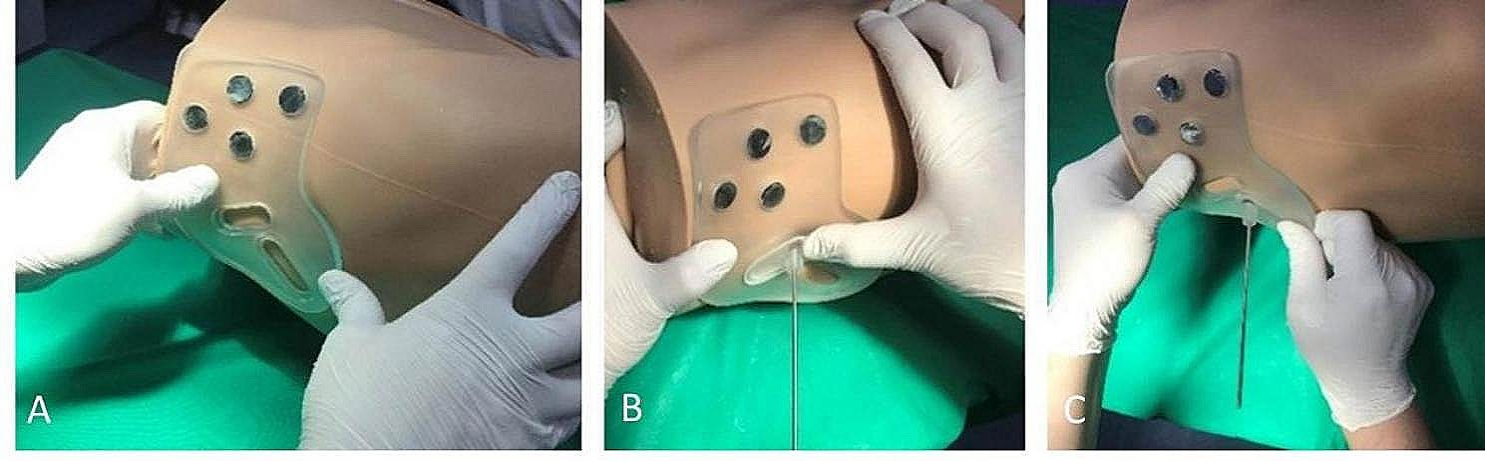
Matching the main body of the guiding template with the external fiducial markers (A), inserting the needle for the suprapubic (B) and sacroiliac screws (C) respectively after embedding the corresponding guiding director into the main body of the guiding template

The Surgeons drilled a 2.5 mm guiding needle into the bone within the guiding director. The depth of the drilling was controlled based on the preoperative recorded virtually planned screw length. The depth grading was seen on the drillbit which removed the need for consistent fluoroscopic confirmation. The premeasured (from preoperative scanning) cannulated screw was then inserted along the guiding needle (Fig. 4) into the virtually planned screw channel. After screw insertion, one static C-arm fluoroscopic image confirmed screw position in inlet, outlet, and lateral views.

### Participants and tasks

**Fig. 5:**
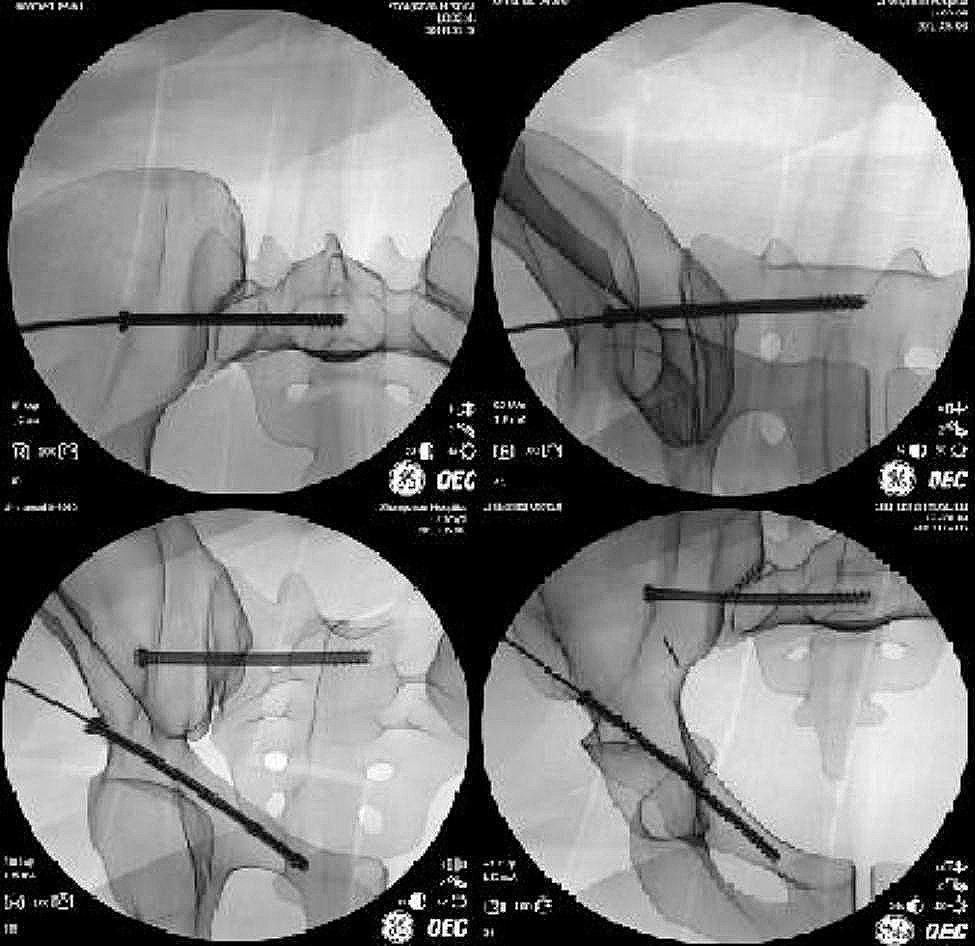
Images of intraoperative C-arm fluoroscopy after insertion of screws assisted by the guide template in phantom experiment

Nine expert fellowship-trained Attending Orthopaedic Surgeons with 5–10 years of experience were recruited. All of them have completed over 200 cases of pelvis fractures. Five Surgeons participated in Phase 1 on five pelvic high-fidelity phantoms and another 4 Surgeons in Phase 2 on cadaveric specimen. Each Orthopaedic Surgeon inserted one anterograde suprapubic screw and one sacroiliac screw in each pelvic phantom model (Fig. 4). Intraoperative C-arm fluoroscopy was performed immediately after screw insertion in Phase 1 only, and the images were saved (Fig. 5).

Following the completion of the experiment, the five participating Attending Orthopedic Surgeons were requested to complete a Likert scale questionnaire to express their acceptability using the guiding template. The questionnaire was designed using a modified Delphi method after three rounds prior to finalization incorporating advice and feedback from three expert Orthopedic Surgeons independent from the study. The questionnaire reflected face, content and concurrent validation of the guiding templates.

### Phase 2: cadaveric translation

**Fig. 6:**
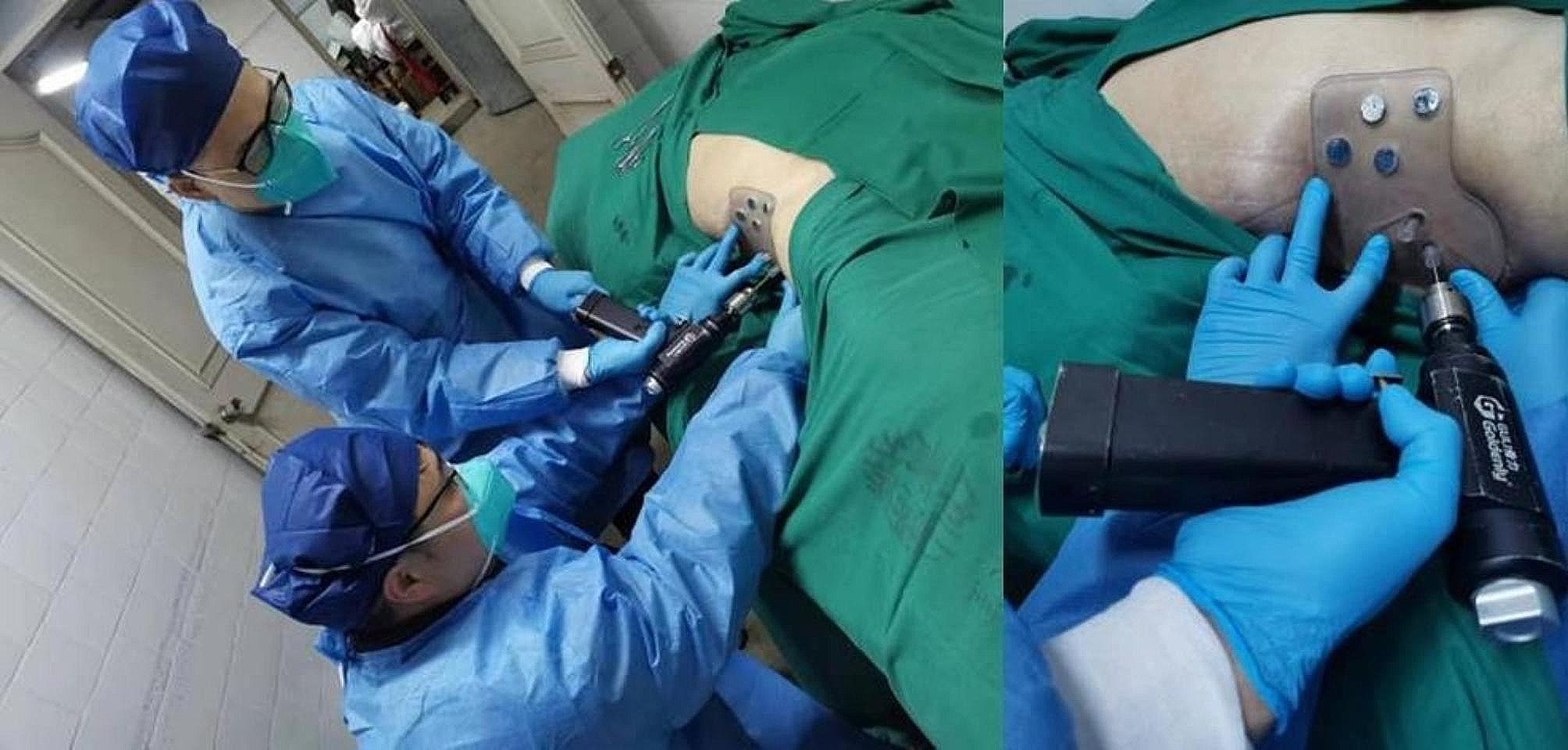
Drilling the guiding needle along the guiding director with the assistance of fixing the guiding template on cadavers

In order to assess the feasibility of our guiding template, cadaveric experiments were additionally conducted. The study was conducted with the approval of the institutional ethics committee, and two intact fresh frozen male cadavers without any pelvic fractures or pathologies observed in CT images were included. For educational and scientific research purposes, written informed consent was obtained from the immediate family members of the deceased. The format of the informed consent form followed the guidelines of the China Organ Donation Management Center. The methods of this study follow the CACTUS guidelines, which provide guidance for anatomical studies and contribute to the quality of research methods [12]. The cadavers were provided by the Department of Human Anatomy and Histoembryology, Shanghai Jiao Tong University. The type of study was anatomical and surgical and the cadavers were exposed only to radiation. There was no pathological evaluation of cadaveric specimens in this investigation, and the entire study was conducted by four expert Attending Orthopaedic Surgeons, who performed bilateral percutaneous anterograde suprapubic screw and sacroiliac screw fixation using the individualized 3D printed guiding template. One Surgeon stabilized the guiding template, while the other Surgeon inserted a guiding needle along the guiding director to insert the screw. The entire operative process was performed without the assistance of C-arm fluoroscopy (Fig. 6).

After the postoperative CT scans, the position of the inserted screws were assessed compared to preoperative planned screw channels. Additionally, the deviation between the position of the postoperative actual screw and the preoperative virtually planned screw was measured following the registration of both preoperative and postoperative 3D pelvic reconstruction models. Likewise, the four orthopedic surgeons were requested to complete the same Likert scale questionnaire.

### Postoperative accuracy evaluation

**Fig. 7:**
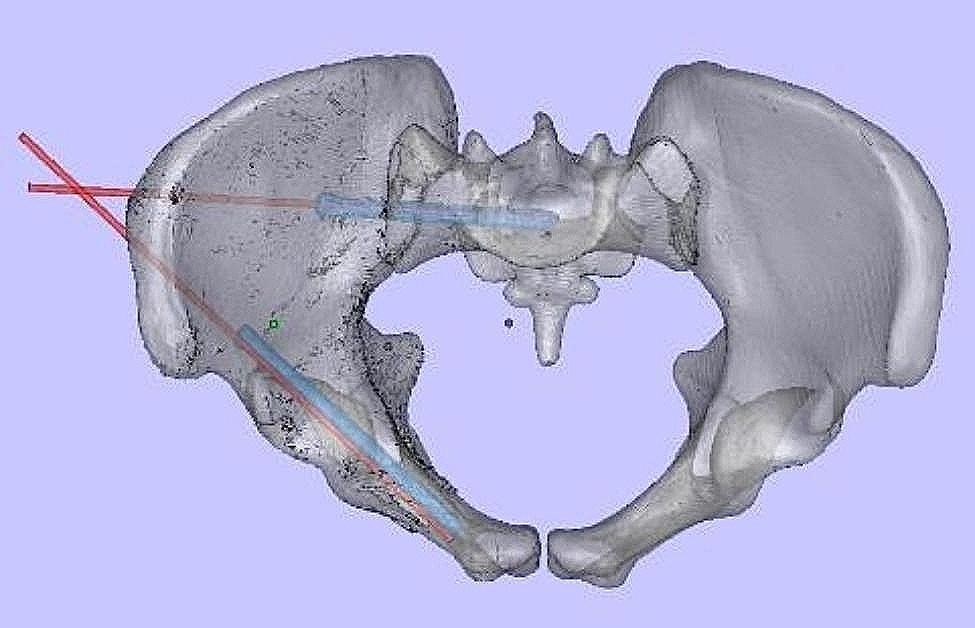
Registration of the 3D models of the preoperative planned screw path and postoperative inserted screw in phantom experiment

**Fig. 8:**
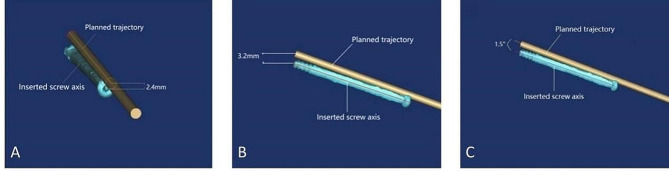
Comparison of post-operative actually inserted screw position with pre-operative planned screw channel (A: Mean longitudinal deviation of bony entry point; B: Mean longitudinal deviation of screw tip; C: Mean angular deviation of 3D space angle)

Following the surgical procedure, a CT scan was conducted to assess the position of the inserted screw. Subsequently, a 3D reconstruction of the postoperative pelvis was performed. By registering the preoperative and postoperative 3D virtual pelvic reconstruction models (Fig. 7), the discrepancies between the final positions of the actual postoperative screw against the preoperatively planned screw were measured. These three quantitative discrepancies consisted of (i) bony entry point deviation, (ii) tip point deviation, and (iii) 3D spatial angle differences (Fig. 8).

### Likert scale questionnaire on acceptability, satisfaction and validity

Six questions were posed to the surgeons after completing the simulation exercise of using the 3D printed guiding template using a Likert scale as follows: 5 = strongly agree, 4 = agree, 3 = neutral, 2 = disagree, 1 = strongly disagree. The questions determined the face (to determine extent of realism of using the guiding template on pelvic models), content (to determine the extent of realism of performing the operation using the guiding template) and concurrent validity (to determine the extent of reality from performing on phantom models to cadaveric specimen using the guiding templates).

### Statistical analysis

Data in this pilot study was treated as non-parametric. Mean longitudinal and angular deviations (alongside standard deviation) from pre-operative scanning and post-operative actual screw placement were calculated. Median scores alongside their interquartile ranges were calculated for the Likert scales. Statistical significance was calculated using Mann-Whitney U test and set as *p* < 0.05.

### Ethical approval

was obtained through the Shanghai Jiao Tong University Affiliated Sixth People’s Hospital, Shanghai without objections for usage of the CT scanner or cadaveric specimen. No person was exposed to the radiation from scanning. The cadaveric specimen were already in use for purposes of surgical training but the pelvic anatomy remained intact at the the time of this experiment.

## Results

In Phase 1, a total of 10 screws consisting of five anterograde suprapubic and sacroiliac screws each were successfully inserted using the guiding template. The 3D printed guiding template did not only exhibit secure alignment onto the pelvic phantom models but also maintained stability during the entire process of guiding needle insertion. Postoperative CT scans confirmed that all screws were positioned safely within the bony cortex and safe channels. Specifically, the suprapubic screws did not penetrate into the acetabular joint or extend beyond the pubic bony cortex. Similarly, the sacroiliac screws did not penetrate into the sacral hiatus, sacral canal, or extend beyond the front of the sacrum (Fig. 5).

### Accuracy

Table 1 showed results from pooled data whereas Table 2 showed results from individual attempts.

**Table 1:** Pooled data of the spatial difference between the preoperative virtually planned and postoperative actually inserted screws on phantom models

Phase 1: phantom models	MLD bony entry point(mm)	MLD screw tip (mm)	MAD (deg)
Pooled 5 attempts on suprapubic screws	3.32 ± 0.30	3.56 ± 0.86	1.34 ± 0.54
Pooled 5 attempts on sacroiliac screws	2.34 ± 0.35	2.68 ± 0.48	1.16 ± 0.27
Pooled 10 attempts of screw insertion	2.83 ± 0.60	3.12 ± 0.81	1.25 ± 0.41

Key: MLD – mean longitudinal deviation; MAD – mean angular deviation

**Table 2:** Detailed data of the spatial difference between the pre-operative virtually planned and post-operative actually inserted screws in phantom experiment

Phase 1: Phantom model	Suprapubic screws			Sacroiliac screws			
	MLD bony entry point(mm)	MLD screw tip (mm)	MAD (deg)	MLD bony entry point(mm)	MLD screw tip (mm)		MAD (deg)
1	3.4	3.8	1.3	2.8	2.4	1.0	
2	3.2	3.6	1.5	2.1	2.0	0.8	
3	3.0	3.6	1.4	2.5	3.0	1.2	
4	3.2	2.2	0.5	1.9	2.8	1.3	
5	3.8	4.6	2.0	2.4	3.2	1.5	

Key: MLD – mean longitudinal deviation; MAD – mean angular deviation

### Acceptability and validity

The results of the Likert scale questionnaire revealed that the usage of 3D printed guiding templates applied on the pelvic phantom models demonstrated acceptability, satisfaction and validity (Table 3).

**Table 3:** Results of the Likert scale questionnaire on using the guiding template

Statement	Median score	
	Phantom experiment	Cadaveric experiment
The biomimetic pelvic phantom models are high-fidelity as they highly simulate and reflect actual human anatomy	5	Not applicable
The guiding template is easy to use and fits with the pelvic specimens	4	4
Compared to traditional methods, this 3D printed guiding template was more helpful to assist with percutaneous pelvic screw insertion	4	4
This guiding template will be applicable in real surgical procedures	4	5
You are willing to use this guiding template on patients	4	4
You are willing to recommend this guiding template to your colleagues	4	4

Key: IQR – interquartile range; 5 = strongly agree, 4 = agree, 3 = neutral, 2 = disagree, 1 = strongly disagree

### Phase 2: cadaveric translation

**Fig. 9:**
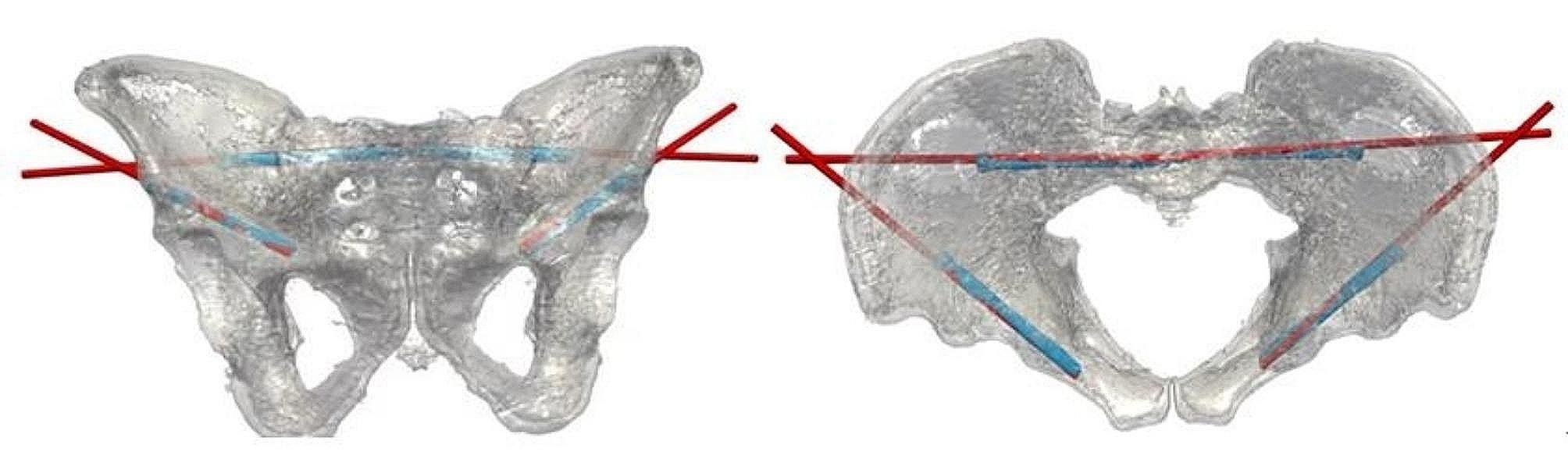
Registration of the 3D scanning models of the preoperative planned screw path and postoperative inserted screw in cadaveric specimen of Phase 2

In the cadaveric experiment, a total of eight screws were successfully inserted into two cadavers, comprising of four anterograde suprapubic and sacroiliac screws each. With the assistance of a surgical assistant, the stability between the cadaveric pelvis and the guiding template was effectively maintained throughout the operation. Postoperative CT scans confirmed that all screws were positioned safely within the bony cortex (Fig. 9).

### Accuracy

Table 4 showed results from pooled data whereas Table 5 showed results from individual attempts.

**Table 4:** Pooled data of the spatial difference between the preoperative virtually planned and postoperative actually inserted screws on cadaveric specimen

Phase 2: Cadaveric specimen	MLD bony entry point(mm)	MLD screw tip (mm)	MAD (deg)
Pooled 4 attempts on suprapubic screws	3.85 ± 0.73	3.53 ± 0.92	1.53 ± 0.76
Pooled 4 attempts on sacroiliac screws	3.25 ± 0.89	3.40 ± 0.59	1.20 ± 0.29
Pooled 8 attempts of screw insertion	3.55 ± 0.82	3.46 ± 0.72	1.36 ± 0.56

Key: MLD – mean longitudinal deviation; MAD – mean angular deviation

**Table 5:** Detailed data of the spatial difference between the preoperative virtually planned and postoperative actually inserted screws in cadaveric specimen for both literalities

Phase 2: Cadaveric specimen		Suprapubic screws			Sacroiliac screws		
		MLD bony entry point(mm)	MLD screw tip (mm)	MAD (deg)	MLD bony entry point(mm)	MLD screw tip (mm)	MAD (deg)
1	**Left**	3.5	4.5	2.2	3.1	3.5	0.8
	**Right**	4.3	2.3	2	2.1	2.9	1.2
2	**Left**	3	3.8	1.4	4.2	3	1.3
	**Right**	4.6	3.5	0.5	3.6	4.2	1.5

Key: MLD – mean longitudinal deviation; MAD – mean angular deviation

## Discussion

### Principal findings

All screws were successfully inserted without any perforation of the far bony cortex or intraarticularly. Furthermore, we compared the preoperative planned screw positions with the actual postoperative positions after scanning. The pooled results indicated a mean longitudinal deviation of 2.83 ± 0.60 mm at the bony entry point of the screw trajectory and 3.12 ± 0.81 mm at the screw tip, as well as a mean angle deviation of 1.25 ± 0.41° in Phase (1) The pooled results were mean longitudinal deviation at the bony entry point of 3.55 ± 0.82 mm and 3.46 ± 0.72 mm at the screw tip, as well as a mean angular deviation of 1.36 ± 0.56° in Phase (2) Expert Surgeons also confirmed a high acceptability, satisfaction and validity.

These results reflected a relatively high level of accuracy on both pelvic models. This accuracy is comparable to that achieved by navigation systems or traditional guiding templates used in pelvic percutaneous screw fixation. The participating expert Surgeons also agreed or highly agreed on its applicability within clinical practice.

### Current options for guiding solutions

The percutaneous placement of pelvic channel screws offers advantages such as minimal tissue invasion and fast post-operative recovery. However, its wide application is limited due to high precision requirements [13–15]. Compared to traditional fluoroscopy solutions, the guiding template solution requires a volumetric imaging in the necessary steps, which can lead to increased radiation for patients. But in most cases, we also perform volumetric imaging on patients to better display the fracture when using traditional fluoroscopy solution. Repeated intraoperative fluoroscopy leads to more radiation exposure for clinicians and patients compared to the guiding template solution.

Various assisting technologies have been developed for minimally invasive screw placement in the pelvis, including 2D fluoroscopy navigation [16], real-time 3D CT navigation [17], infrared 3D navigation [18], and augmented reality combined navigation systems [19]. The navigation system is highly competitive in assisting percutaneous screw implantation in the pelvis. It can calibrate the spatial position of the pelvis and surgical instruments after non-invasive registration and implant screws through a planned path with minimal error. But in most cases the tracker should be firmly fixed to the pelvis through invasion and navigation systems are only equipped in a few large medical centers.

### Limitations of alternative 3D guides

Our templated guides offer a plausible and affordable alternative solution for assisting percutaneous screw fixation of pelvic fractures [11, 20, 21]. However, the currently reported clinical application of 3D printed guiding templates primarily relies on the bone surface landmarks at the surgical site for matching. This requires Surgeons to separate the soft tissue from the bone surface to expose the bony cortex and align it with the guiding template. Unfortunately, this approach often leads to extensive soft tissue dissection, additional surgical trauma and risk of complications. Furthermore, incomplete separation of soft tissue on the bony surface during the surgery can decrease the accuracy of the matching process, particularly in the pelvic region where the soft tissue is thick and is deep.

### Benefits of our 3D printed personalized guiding templates compared to alternative options

To address the aforementioned issues, some researchers have explored the use of guiding templates based on external markers, eliminating the need for intraoperative exposure of the bony surface. For instance, Hu et al. [10]. utilized a 3D printed guiding template for percutaneous vertebroplasty, placing it on the skin and aligning it with the preoperatively marked position. Yin et al. [22]. employed a 3D printed guiding template with cutaneous markers for closed fixation of scaphoid fractures, enabling single-step placement. Zhang et al. [23]. applied a guiding template with skin markers in sacral nerve stimulation in urology, successfully guiding the needle to puncture the posterior sacral hiatus. This approach resulted in significantly reduced operation time, puncture attempts, and fluoroscopy usage compared to traditional techniques.

While these studies focused on the feasibility of using external guiding templates in these procedures, the accuracy of these templates was not investigated. To the best of our knowledge, the design and application of guiding templates based on skin markers for pelvic surgeries have not been reported in the literature. Therefore, this study represents the attempt to employ such novel guiding templates for pelvic fracture fixation, aiming to preliminarily assess their feasibility in clinical practice.

Takao et al. [17]. reported the use of a CT-3D-fluoroscopy matching navigation system for percutaneous iliosacral screw insertion, where the mean deviation between the planned and inserted screw positions was 2.5 mm at the bony entry point and 2.2 mm at the screw tip. In our previous study, we presented an augmented reality (AR)-based navigation system for percutaneous sacroiliac screw insertion, which showed a mean deviation of 2.7 ± 1.2 mm at the bony entry point, 3.7 ± 1.1 mm at the screw tip, and a mean angular deviation of 2.9° ± 1.1° [19]. However, this novel personalized guiding template introduced in our study is much easier to use intraoperatively, and the matching process of the guide-skin interface before inserting the guidewire and screw only takes 2–3 min, which is significantly shorter than the setup procedure of the navigation systems (10–20 min).

Regarding traditional guiding templates, Yang et al. described the use of a patient-specific external template to guide the insertion of iliosacral screws. The mean deviation between the planned and actual screw positions was 1.52 ± 0.48 mm at the bony entry point and 2.75 ± 1.0 mm at the screw tip, with a mean deviation angle of 1.73 ± 0.80° [24], which is comparable to the accuracy of our guiding template. Zhou et al. also designed a guiding template to assist the implantation of pelvic screws based on pelvic external fixators, where the mean deviation distance and angle between the actual and planned screw positions were 2.6 ± 0.2 mm and 2 ± 0.3° respectively [25]. However, compared to these traditional guides, our guiding template has the advantage of not causing additional damage since it does not require the exposure of the bony surface or the insertion of external fixation for matching purposes.

### Technical advantages of our 3D printed guiding template

The design of our guiding template ensures a high level of stability between the guide and the pelvis during the operation. The guiding director, specially designed for this purpose, not only guides the insertion of the screw (or guiding needle) but also provides support and stabilization to the main body of the guiding template. The length of the guiding director is determined based on the preoperative plan, taking into account the thickness of the soft tissue, so that it can establish contact against the bony surface. The tip of the guiding director is designed with sawtooth patterns to increase friction between it and the bone surface, thereby enhancing stability. By integrating the skin markers and the guiding director into the main body of the guiding template and sequentially pushing the tip of the guiding director against the bone surface, a triangular construct is achieved to maintain stability and control.

### Error analysis and plausible solutions to our guiding template

The main factor contributing to the instability of the guiding template is the drift of the soft tissue near the external markers. This soft tissue drift primarily involves lateral sliding and compression deformation, which can cause changes in the position of the entire guiding template. The lateral sliding of the soft tissue relative to the bone can be mitigated to a large extent by the stabilizing effect from the triangular construct. Nevertheless, if lateral sliding occurs, it may result in a deviation of the bony entry point. Additionally, the compression deformation of the soft tissue introduces some errors. Although we have placed the skin markers near the iliac crest, where the soft tissue thickness is relatively at its thinnest at the pelvis, typically ranging from 5 to 10 mm, compression deformation is still unavoidable. To be certain, soft tissue drift is the main cause of the screw position deviation observed in our study.

In order to minimize errors, we propose the following suggestions. Firstly, it should be noted that obese patients with thicker soft tissue envelope may experience larger margins of error and may not be suitable candidates for our guiding templates. Secondly, ensure that the tip of the guiding director is pressed firmly against the bony cortex. The tip of the guiding director can be designed with saw teeth to enhance the friction between the guide-bone interface, thereby potentially improving its stability. Thirdly, in clinical settings, patient safety should always be ensured. Therefore, despite the assistance provided by our guiding template, intraoperative fluoroscopy may still be necessary to confirm the position of the guiding needle after it has been placed into the bony channel. Our guiding template can significantly reduce the frequency of intraoperative fluoroscopy, serving as an auxiliary technology.

### Limitations, implications and future developments

The application of the guiding templates in high-fidelity biomimetic pelvic phantom models may raise concerns regarding the generalizability of the results. However, it should be noted that these phantom models were specifically designed to closely simulate the pelvic bone and its soft tissues envelope for the purpose of training in pelvic fracture fixation. The bony and soft tissue representations within the phantom models were 3D printed based on data from the Chinese digital human, using biomimetic materials that were manufactured to replicate the tissue strength, elasticity and structural properties observed in cadaveric testing. Furthermore, the results of a questionnaire completed by five expert Attending Orthopaedic Surgeons also indicated that these phantom models provided a comparable experience to real human conditions.

Phase 2 was also conducted to further evaluate the accuracy of the guiding template in cadavers. The results also demonstrated high level of feasibility and accuracy of the our guiding template, and high satisfaction from the surgeons. Secondly, it should be noted that intact pelvic phantom models and cadaveric specimen without fractures were utilized in this study. However, these intact phantom models were specifically designed to simulate pelvic fractures without obvious displacement or those that had undergone acceptable fracture reduction. Therefore, our guiding template can only be applied to stable pelvic fractures without significant fracture displacement, or unstable pelvic fractures that have been stabilized using other methods after close reduction such as external fixation. In our initial study, a virtual reduction system for pelvic and acetabular fractures was developed to improve reduction accuracy and decreases reduction time [26]. In the following study, combination of the reduction system and the guiding template would be highly anticipated. Thirdly, for each patient, it may take 2–3 days and cost 200 dollars to complete the design and printing of the guiding template. However, it is cost effective and provides high accuracy of implant position during the surgical phase, allowing predictable outcomes for the restorative phase.

## Conclusion

Our personalized 3D printed guiding template based on cutaneous fiducial markers demonstrated safe, easier and acceptable percutaneous pelvic screw insertion after phantom model and cadaveric testing. It offers the advantage of avoiding additional tissue damage by eliminating the need to expose the bony surface for matching, distinguishing it from the currently reported individualized guides. It should be pointed out that the drift of soft tissue can cause errors in the accuracy. This guiding template has the potential to serve as a reliable alternative or adjunct to the traditional fluoroscopically-guided method for percutaneous pelvic screw fixation in current clinical practice. Our guide can be particularly beneficial for stable pelvic fractures without significant displacement, unstable pelvic fractures with minimal displacement or those that have already undergone reduction while avoiding excessive dissection, surgical trauma and operative complications.

## Data Availability

All data generated or analysed during this study are included in this published article.

## References

[1] Balbachevsky D, Belloti JC, Doca DG, Jannarelli B, Junior JA, Fernandes HJ, Dos Reis FB (2014). Treatment of pelvic fractures - a national survey. Injury.

[2] Hoskins W, Bucknill A, Wong J, Britton E, Judson R, Gumm K, Santos R, Sheehy R, Griffin X (2016). A prospective case series for a minimally invasive internal fixation device for anterior pelvic ring fractures. J Orthop Surg Res.

[3] Scheyerer MJ, Zimmermann SM, Osterhoff G, Tiziani S, Simmen HP, Wanner GA, Werner CM (2014). Anterior subcutaneous internal fixation for treatment of unstable pelvic fractures. BMC Res Notes.

[4] Zhang R, Yin Y, Li S, Guo J, Hou Z, Zhang Y (2019). Sacroiliac screw versus a minimally invasive adjustable plate for Zone II sacral fractures: a retrospective study. Injury.

[5] Lee CH, Hsu CC, Huang PY. Biomechanical study of different fixation techniques for the treatment of sacroiliac joint injuries using finite element analyses and biomechanical tests. Comput Biol Med 2017, 87:250–710.1016/j.compbiomed.2017.06.007. 10.1016/j.compbiomed.2017.06.007 28618337

[6] Shih YC, Beaubien BP, Chen Q, Sembrano JN (2018). Biomechanical evaluation of sacroiliac joint fixation with decortication. Spine Journal: Official J North Am Spine Soc.

[7] Huang H, Hsieh MF, Zhang G, Ouyang H, Zeng C, Yan B, Xu J, Yang Y, Wu Z, Huang W (2015). Improved accuracy of 3D-printed navigational template during complicated tibial plateau fracture surgery. Australasian Phys Eng Sci Med.

[8] Kaneyama S, Sugawara T, Sumi M, Higashiyama N, Takabatake M, Mizoi K (2014). A novel screw guiding method with a screw guide template system for posterior C-2 fixation: clinical article. J Neurosurg Spine.

[9] Omori S, Murase T, Kataoka T, Kawanishi Y, Oura K, Miyake J, Tanaka H, Yoshikawa H (2014). Three-dimensional corrective osteotomy using a patient-specific osteotomy guide and bone plate based on a computer simulation system: accuracy analysis in a cadaver study. Int J Med Rob + Comput Assist Surgery: MRCAS.

[10] Hu P, Lin J, Xu J, Meng H, Su N, Yang Y, Fei Q. Three-Dimensional Printing Guide Template assisted Percutaneous Vertebroplasty (PVP). J Visualized Experiments: JoVE. 2019;152. 10.3791/60010. 10.3791/60010 31680679

[11] Liu Y, Zhou W, Xia T, Liu J, Mi BB, Hu LC, Shao ZW, Liu GH (2018). Application of the Guiding Template designed by three-dimensional Printing Data for the insertion of Sacroiliac screws: a new clinical technique. Curr Med Sci.

[12] Mantica G, Leonardi R, Diaz R, Malinaric R, Parodi S, Tappero S, Paraboschi I, Álvarez-Maestro M, Yuen-Chun Teoh J, Garriboli M, et al. Reporting ChAracteristics of cadaver training and sUrgical studies: the CACTUS guidelines. Int J Surg (London England). 2022;101106619. 10.1016/j.ijsu.2022.106619. 10.1016/j.ijsu.2022.106619 35429658

[13] Docquier PL, Paul L, Cartiaux O, Banse X (2009). Registration accuracy in computer-assisted pelvic surgery. Comput Aided Surgery: Official J Int Soc Comput Aided Surg.

[14] Lázaro Gonzálvez A, Martínez Reina J, Cano Luis P, Jiménez Baquero J, Sueiro Fernández J, Giráldez Sánchez MA (2016). Is cannulated-screw fixation an alternative to plate osteosynthesis in open book fractures? A biomechanical analysis. Injury.

[15] Yu KH, Hong JJ, Guo XS, Zhou DS (2015). Comparison of reconstruction plate screw fixation and percutaneous cannulated screw fixation in treatment of tile B1 type pubic symphysis diastasis: a finite element analysis and 10-year clinical experience. J Orthop Surg Res.

[16] Liu HS, Duan SJ. Robot-assisted percutaneous screw placement combined with pelvic internal fixator for minimally invasive treatment of unstable pelvic ring fractures. 2018, 14(5):e1927.10.1002/rcs.1927. 10.1002/rcs.1927 PMC6175104 29920914

[17] Takao M, Nishii T, Sakai T, Yoshikawa H, Sugano N (2014). Iliosacral screw insertion using CT-3D-fluoroscopy matching navigation. Injury.

[18] Richter PH, Gebhard F, Dehner C, Scola A (2016). Accuracy of computer-assisted iliosacral screw placement using a hybrid operating room. Injury.

[19] Wang H, Wang F, Leong AP, Xu L, Chen X. Precision insertion of percutaneous sacroiliac screws using a novel augmented reality-based navigation system: a pilot study. 2016, 40(9):1941–710.1007/s00264-015-3028-8. 10.1007/s00264-015-3028-8 26572882

[20] Chen B, Zhang Y, Xiao S, Gu P, Lin X (2012). Personalized image-based templates for iliosacral screw insertions: a pilot study. Int J Med Rob + Comput Assist Surgery: MRCAS.

[21] Chen H, Wang G, Li R, Sun Y, Wang F, Zhao H, Zhang P, Zhang X (2016). A novel navigation template for fixation of acetabular posterior column fractures with antegrade lag screws: design and application. Int Orthop.

[22] Yin HW, Xu J, Xu WD (2017). 3-Dimensional Printing-assisted percutaneous fixation for Acute Scaphoid fracture: 1-Shot Procedure. J Hand Surg.

[23] Zhang J, Zhang P, Wu L, Su J, Shen J, Fan H, Zhang X (2018). Application of an individualized and reassemblable 3D printing navigation template for accurate puncture during sacral neuromodulation. Neurourol Urodyn.

[24] Yang F, Yao S, Chen KF, Zhu FZ, Xiong ZK, Ji YH, Sun TF, Guo XD. A novel patient-specific three-dimensional-printed external template to guide iliosacral screw insertion: a retrospective study. 2018, 19(1):39710.1186/s12891-018-2320-3. 10.1186/s12891-018-2320-3 PMC6234543 30424773

[25] Zhou K, Tao X, Pan F, Luo C, Yang H (2022). A novel patient-specific three-Dimensional Printing Template based on external fixation for pelvic screw insertion. J Invest Surgery: Official J Acad Surg Res.

[26] Wang H, Wang F, Newman S, Lin Y, Chen X, Xu L, Wang Q (2016). Application of an innovative computerized virtual planning system in acetabular fracture surgery: a feasibility study. Injury.

